# A Method for Visualization of Incoming Adenovirus Chromatin Complexes in Fixed and Living Cells

**DOI:** 10.1371/journal.pone.0137102

**Published:** 2015-09-02

**Authors:** Tetsuro Komatsu, Denis Dacheux, Florian Kreppel, Kyosuke Nagata, Harald Wodrich

**Affiliations:** 1 Microbiologie Fondamentale et Pathogénicité, MFP CNRS UMR 5234, Université de Bordeaux, Bordeaux 33076, France; 2 Department of Infection Biology, Faculty of Medicine, University of Tsukuba, Tsukuba 305–8575, Japan; 3 Bordeaux INP, MCMP, UMR 5234, Bordeaux 33000, France; 4 Department of Gene Therapy, Ulm University, Ulm 89081, Germany; Karolinska Institutet, SWEDEN

## Abstract

Inside the adenovirus virion, the genome forms a chromatin-like structure with viral basic core proteins. Core protein VII is the major DNA binding protein and was shown to remain associated with viral genomes upon virus entry even after nuclear delivery. It has been suggested that protein VII plays a regulatory role in viral gene expression and is a functional component of viral chromatin complexes in host cells. As such, protein VII could be used as a maker to track adenoviral chromatin complexes in vivo. In this study, we characterize a new monoclonal antibody against protein VII that stains incoming viral chromatin complexes following nuclear import. Furthermore, we describe the development of a novel imaging system that uses Template Activating Factor-I (TAF-I/SET), a cellular chromatin protein tightly bound to protein VII upon infection. This setup allows us not only to rapidly visualize protein VII foci in fixed cells but also to monitor their movement in living cells. These powerful tools can provide novel insights into the spatio-temporal regulation of incoming adenoviral chromatin complexes.

## Introduction

Adenovirus (Ad) is a non-enveloped virus with a linear double-stranded DNA genome. In the virion, the Ad genome forms a chromatin-like structure with viral basic core proteins, protein V, VII, and polypeptide X/mu [1]. Among them, protein VII is the major DNA binding protein and shown to introduce superhelical turns into DNA, similar to cellular histones [2]. Protein VII is thought to largely remain associated with the viral genome, at least during the first hours of infection (including its nuclear import), although how long this association lasts is subject to debate [3]. Genome association after nuclear import is supported by several biochemical assays [4], including chromatin immunoprecipitation (ChIP) assays [5–8], and microscopy (see below). Furthermore, we have reported using reconstituted protein VII-DNA complexes that protein VII can enhance gene expression in *cis* [6], indicating a functional role in the regulation of viral gene expression in the nucleus. In contrast to protein VII, core protein V appears to be lost before nuclear import of the genomes [9]. The fate of polypeptide X/mu remains to be determined. Thus, during the first hours after nuclear import, the Ad chromatin complex is composed of at least genomic viral DNA and protein VII.

The fate of incoming Ad chromatin complexes after nuclear import remains elusive. Immunofluorescence (IF) analyses using protein VII-specific antibodies labeled discrete nuclear puncta, presumably incoming Ad chromatin complexes [8]. Additional imaging approaches include direct detection of the viral DNA using fluorescence *in situ* hybridization (FISH) but suffer from the harsh specimen preparation [10]. Alternative less invasive methods for labeling viral genomes have been reported, such as AdLite virus, a viral particle containing an indirectly GFP-labeled genome, which was generated based on the combination of the inserted *teto* sequence and GFP-tagged TetR protein [11]. This system succeeded in visualizing the cytoplasmic transport of the viruses but failed to detect intranuclear genomes [11]. Most recently, Greber and co-workers reported a novel approach that involves labeling of viral DNA with “clickable” nucleoside analogs such as 5-ethynyl-2’-deoxycytidine (EdC) [12]. This technique allowed the visualization of incoming Ad genomes and confirmed that the vast majority of labeled Ad genomes in nuclei were protein VII-positive. This indicates that protein VII can be used as a surrogate marker to detect incoming viral chromatin complexes [12]. IF analysis using anti-protein VII antibodies is simple and reliable, but this technique is restricted to use on fixed cells.

Live-cell imaging analyses have many advantages over the study of fixed cells. To the best of our knowledge, no system to monitor individual Ad chromatin complexes in real-time has been established. We previously identified several cellular factors that can remodel the Ad chromatin-like structure *in vitro* [13–15]. One of these factors, Template Activating Factor (TAF)-I/SET, was found to be associated with incoming viral genomes through the interaction with protein VII in infected cells [5,16]. Knockdown of TAF-I resulted in reduction of early viral gene expression [6,16], suggesting a critical role of the factor in regulating viral chromatin functions early in infection. Our previous IF analyses also indicated that TAF-I co-localized with protein VII puncta [16], which was confirmed by ChIP analyses [5,6]. Taken together, accumulating evidence suggests that TAF-I is a functional component of Ad chromatin complexes during early phases of infection and could be used as a marker of incoming nuclear viral chromatin complexes.

In this study we establish novel experimental approaches to image incoming Ad chromatin complexes. First we characterize a novel monoclonal antibody against protein VII that recognizes incoming Ad chromatin complexes upon nuclear import using IF analyses. Secondly, we develop an innovative imaging setup using fluorescent protein-tagged TAF-I. This technique allows us to visualize viral chromatin complexes without using antibodies and monitor incoming Ad chromatin complexes in living cells in real-time. This work provides a unique and powerful set of experimental tools to further our understanding of Ad chromatin regulation in early phases of infection.

## Materials and Methods

### Cells and viruses

U2OS (ATCC #HTB-96) and HEK293 (ATCC #CRL-1573) cells were maintained in DMEM Glutamax (Life Technologies) supplemented with 10% of fetal calf serum (FCS). Recombinant human adenovirus type 5 (Ad5) and replication-deficient E1-deleted GFP-expressing vector (Ad5-GFP) were amplified and purified as described previously [17,18]. Labeling of viral particles with Alexa dyes was done using a small-scale protein-labeling kit (Life Technologies) as described previously [17,19]. The transfection of plasmids was done using Lipofectamine 2000 (Life Technologies) according to the manufacturer’s protocol.

A recombinant Ad vector expressing His-tagged protein VII (HAdV-C5-His-protein VII) was generated as follows. The coding sequence of protein VII (nt 16287–16475 in GenBank:AY339865.1) was amplified by PCR from the plasmid pGS66 [20] and subcloned into pTrcHisA (Invitrogen) to N-terminally attach a 6xHis-tag followed by an enterokinase cleavage site. The coding sequence was excised by NcoI/EcoRI and subcloned into an expression plasmid derived from pCMVbeta (Clontech) to construct an hCMV promoter-driven expression cassette. This expression cassette was subcloned into the E1 region of a ΔE1ΔE3 human Ad5-based replication-defective vector genome in pCV100 (data not shown). To rescue the corresponding Ad vector, the vector genome was released by digestion with SwaI and transfected into N52.E6 cells [20]. The vector was amplified on N52.E6 cells and purified by CsCl gradient. The vector was desalted and titrated by a DNA-based slot-blot procedure [21].

### Purification of His-tagged protein VII

HEK293 cells were infected with HAdV-C5-His-protein VII and cultured for 36 hours. His-tagged protein VII was purified under denaturing conditions from infected cells using Protino Ni-TED Packed Columns (MACHEREY-NAGEL) according to the manufacturer’s protocol.

### Antibodies

Rabbit anti-protein VII was kindly provided by D. A. Engel (University of Virginia School of Medicine) [7]. Rat anti-protein VII and mouse anti-TAF-Iβ (KM1720) antibodies are described elsewhere [5,22].

Monoclonal antibodies were raised against purified His-tagged protein VII in mice using the protocol as previously described [23]. Hybridomas were cloned by limiting dilution and screened by immunofluorescence on infected cells. One monoclonal antibody obtained from hybridoma clone #2–14 was characterized in this study.

### Plasmids

The expression vectors for EGFP-tagged wildtype TAF-Iβ and its PME mutant (pEGFP-C1-TAF-Iβ and pEGFP-C1-TAF-IβPME) were generous gifts from K. Kato and K. Kajitani (University of Tsukuba). The expression vector for histone H2B-tdiRFP (pCAG-H2B-tdiRFP-IP) was obtained from M.-E. Torres-Padilla’s laboratory (Université de Strasbourg) *via* Addgene (plasmid #47884) [24].

### Immunofluorescence analysis

Indirect IF analyses were done as described previously [17,18,23] with minor modifications. Briefly, cells grown on coverslips were washed with PBS (phosphate-buffered saline) and fixed with 4% EM-grade paraformaldehyde (PFA) in PBS. Alternatively, cells were first incubated with Transport buffer (20 mM HEPES, 110 mM potassium acetate, and 2 mM magnesium acetate, pH 7.5) containing 0.5% Triton X-100 for pre-extraction and then fixed with 4% EM-grade PFA in PBS. Cells were permeabilized with 0.5% Saponin in PBS and blocked with IF-buffer (10% fetal calf serum and 0.2% Saponin in PBS). Primary and secondary antibodies (Alexa Fluor-labeled antibodies against mouse/rabbit/rat IgG, Life Technologies) were applied to the coverslip in IF-buffer for 1 h each. Cells were mounted in DAKO mounting media containing DAPI, and were analyzed by a Leica SP5 confocal microscope. Confocal stacks were taken every 0.3 μm, and images were processed using ImageJ and presented as maximum intensity projections.

### Live-cell imaging

Details for the imaging setup are described elsewhere [19]. Approximately 1x10^5^ U2OS cells were transfected with 0.5 μg of pEGFP-C1-TAF-Iβ using Lipofectamine 2000. Then, cells were seeded in ibidi μ-Slide VI^0.4^ (Ibidi) and either mock-infected or infected with Alexa594-labeled Ad5-GFP. Before starting imaging, the medium was replaced by CO_2_-independent medium (Life Technologies) supplemented with 10% FCS and 4 mM Glutamax (Life Technologies). For imaging, we chose cells showing modest expression levels of EGFP-TAF-Iβ to obtain better image qualities. Images were acquired on a Leica spinning-disk microscopy system (x100 objective) equipped with an incubation chamber at 37°C. For each color channel, frames were taken every 3 s and assembled into movies using MetaMorph software.

### Western blotting

To prepare cell lysates, cells were resuspended in Pierce IP Lysis buffer (Thermo scientific) containing 1 mM PMSF and then sonicated. Lysates were subjected to SDS-PAGE and proteins were transferred to nitrocellulose membranes (Thermo scientific). Membranes were blocked with 5% skimmed milk/TBS (Tris-buffered saline) and then incubated with indicated primary antibodies. Primary antibodies were detected using HRP-conjugated secondary antibodies (Interchim). The blots were developed using Immobilon Western Chemiluminescent HRP substrate (millipore).

### Ethics statement

All animal experiments were performed in accordance with institutional guidelines as determined by the Service Commun des Animaleries de l’Université Bordeaux (approval number A33-063-916), which specifically approved the study.

## Results

**Fig 1:**
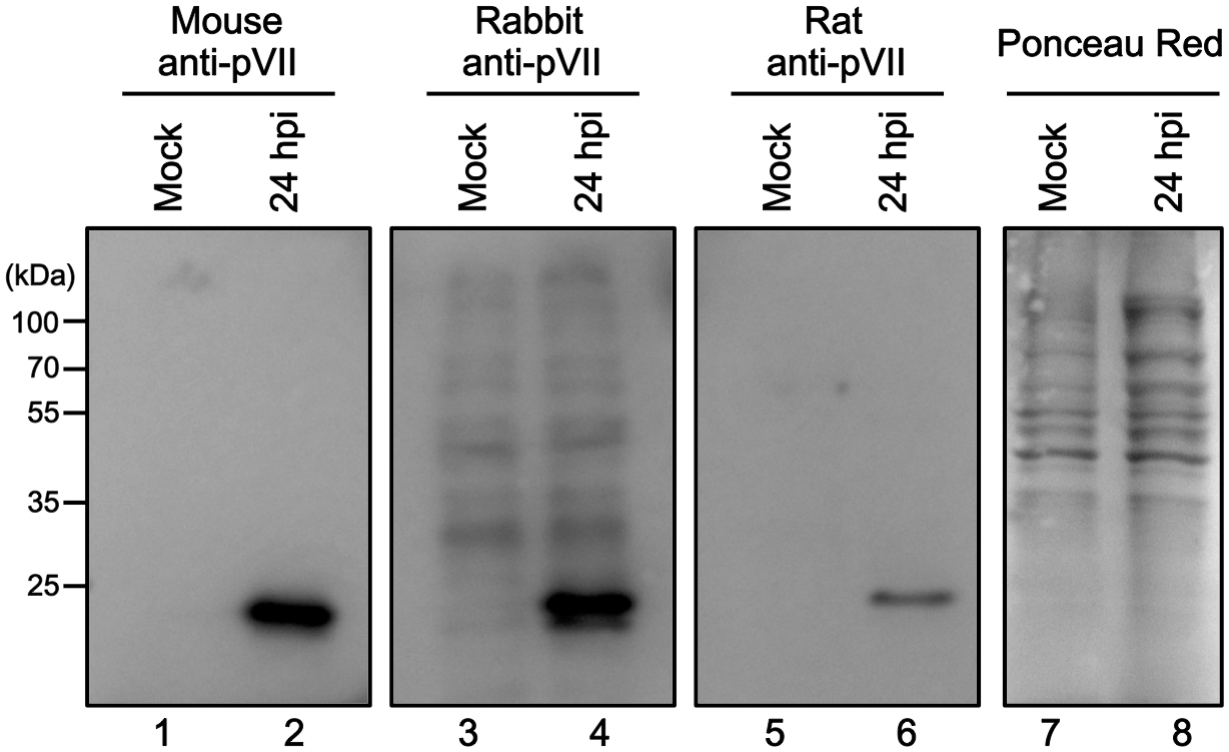
Western blotting using anti-protein VII antibodies. U2OS cells were either mock-infected (lanes 1, 3, 5, and 7) or infected with Ad5 (lanes 2, 4, 6, and 8) and collected at 24 hpi. Cell lysates were prepared and subjected to 12% SDS-PAGE. Proteins were transferred to membranes and subjected to western blot analyses using mouse (lanes 1 and 2), rabbit (lanes 3 and 4), or rat anti-protein VII antibodies (lanes 5 and 6) or Ponceau Red staining as loading control (lanes 7 and 8).

To develop new imaging setup for Ad chromatin complexes, monoclonal antibodies against purified His-tagged recombinant protein VII were raised in mice. The hybridoma clone #2–14 was selected for this study. Western blot analyses were done to examine the specificity of the monoclonal antibody by comparing it with two other rabbit and rat anti-protein VII antibodies available (Fig 1 and S1 Fig). Cell lysates were prepared from mock-infected and Ad5-infected cells at 24 hpi (hours post infection), and western blotting was done using all three anti-protein VII antibodies. All of the antibodies detected proteins around 25 kDa that were specific to Ad infection (Fig 1), indicating high specificity and the absence of unspecific binding to other viral and/or cellular proteins.

**Fig 2:**
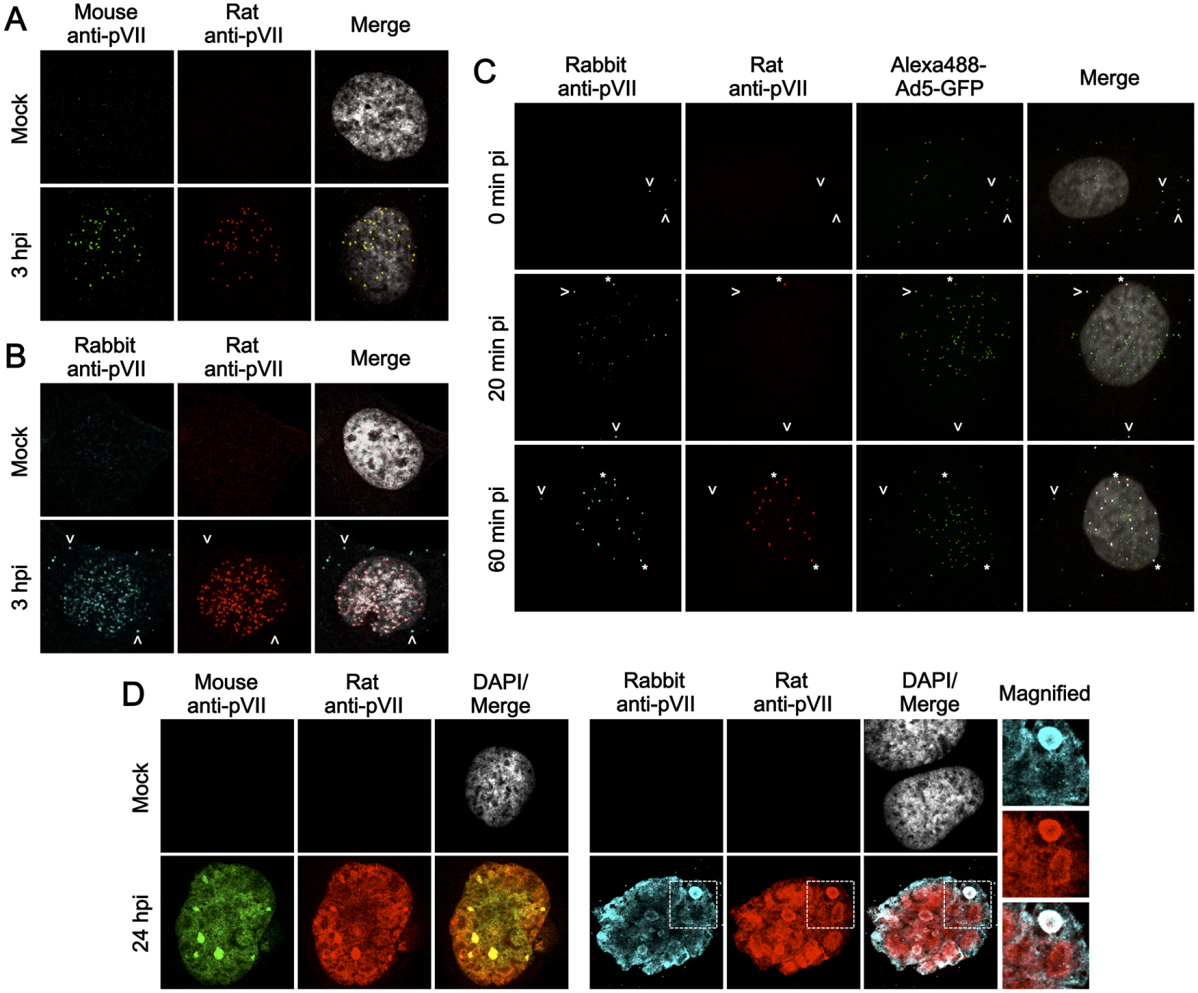
IF analyses using anti-protein VII antibodies. (A) IF analyses with mouse and rat anti-protein VII antibodies. U2OS cells were either mock-infected or infected with Ad5 and at 3 hpi subjected to IF analyses with mouse (green) and rat anti-protein VII antibodies (red). Merged images with DAPI staining (gray) are also shown. (B) IF analyses with rabbit and rat anti-protein VII antibodies. IF analyses were carried out as described in (A) but using rabbit (cyan) and rat anti-protein VII antibodies (red). Arrows indicate cytoplasmic protein VII signals. (C) IF analyses with Alexa-labeled viruses. U2OS cells were first incubated with Alexa488-labeled Ad5-GFP (green) at 4°C for adsorption, and then transferred to 37°C. After 0, 20, and 60 min, cells were collected for IF analyses using rabbit (cyan) and rat anti-protein VII antibodies (red). Asterisks indicate protein VII signals at the nuclear rim. (D) IF analyses using cells at late phases of infection. U2OS cells were either mock-infected or infected with Ad5, and at 24 hpi subjected to IF analyses using either mouse and rat (green and red, left panels) or rabbit and rat anti-protein VII antibodies (cyan and red, right panels). DAPI staining (gray) and merged images were shown for mock and infected cells, respectively. For right panels, magnified images of the regions marked by squares are also shown.

IF analyses were done using the same set of anti-protein VII antibodies described above (Fig 2 and S1 Dataset). At 3 hpi, mock-infected and Ad5-infected U2OS cells were subjected to IF analyses using mouse and rat anti-protein VII antibodies (Fig 2A). Both antibodies labeled the same nuclear puncta, confirming the specificity of the new monoclonal antibody. The same IF analyses were carried out using rabbit and rat antibodies for comparison (Fig 2B). As with the mouse antibody, the nuclear puncta of protein VII were detected by both antibodies. In addition, occasionally cytoplasmic protein VII signals were observed with the rabbit antibody (Fig 2B, arrows) but not the rat or the mouse antibody. To examine whether the cytoplasmic signals were connected to viral particles, IF analyses were done with synchronized infection using Alexa-labeled viruses (Fig 2C). Even immediately after the adsorption step, cytoplasmic signals were only observed with the rabbit antibody, some of which were overlapping with viral particles, suggesting a specific signal (Fig 2C, 0 hpi, arrows). Nuclear puncta of protein VII started to appear at 20 min pi, and the number of nuclear protein VII puncta reached the maximum level around 1 hpi independent of the antibody used (Fig 2C, asterisks, and data not shown). To further compare the antibodies, IF analyses were performed using infected cells at late phases of infection (24 hpi, Fig 2D). While mouse and rat antibodies had similar staining patterns (Fig 2D, left panels), the rabbit antibody stained both similar and distinct subnuclear structures (Fig 2D, right panels). In summary, although their specificities appear somewhat different, all of the protein VII antibodies tested here, including the novel mouse monoclonal antibody, are suitable for the detection of incoming Ad chromatin complexes after nuclear import using IF analyses.

**Fig 3:**
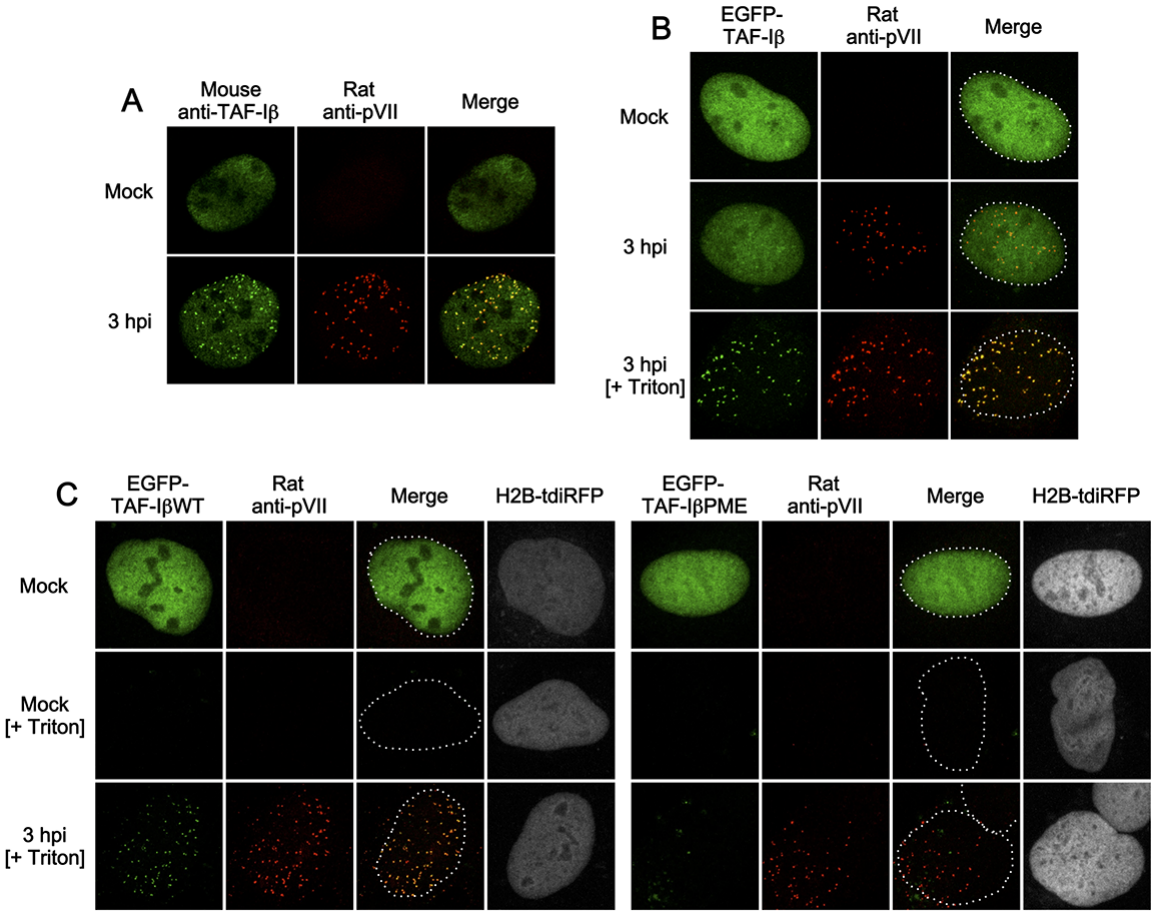
IF analyses using EGFP-TAF-Iβ. (A) IF analyses with anti-TAF-Iβ antibody. U2OS cells were either mock-infected or infected with Ad5. At 3 hpi, cells were subjected to IF analyses using mouse anti-TAF-Iβ (green) and rat anti-protein VII antibodies (red). (B) IF analyses with EGFP-TAF-Iβ. U2OS cells were transiently transfected with the expression vector for EGFP-TAF-Iβ (green) and at 24 hpt (hours post transfection) were either mock-infected or infected with Ad5. At 3 hpi, cells were either immediately fixed (first and second rows) or pre-extracted with Triton X-100 and then fixed (third row, + Triton) and subjected to IF analyses using rat anti-protein VII antibody (red). Nuclear shapes are indicated by dashed lines. (C) IF analyses with TAF-IβPME mutant. U2OS cells were transiently transfected with the expression vectors for either EGFP-TAF-IβWT (left) or PME mutant (right panels), together with the one for histone H2B-tdiRFP (gray), and at 24 hpt IF analyses were carried out as described above.

The cellular protein TAF-I (Fig 3 and S2 Dataset), which co-localizes with protein VII in the nucleus upon Ad infection in HeLa cells [16], was then characterized. The endogenous TAF-Iβ co-localized with protein VII puncta in U2OS cells (Fig 3A). We reasoned that if fluorescent protein-tagged TAF-Iβ behaves like endogenous one, it could be an alternative marker for protein VII puncta in both fixed and living cells. We carried out IF analyses using EGFP-tagged TAF-Iβ and anti-protein VII antibodies (Fig 3B). Similar to the endogenous protein, EGFP-TAF-Iβ showed an even nuclear localization in uninfected cells (Fig 3B, first row). However, we could not observe clear overlap with protein VII puncta in infected cells (Fig 3B, second row) unless cells were pre-extracted using detergent prior to fixation (Fig 3B, third row), suggesting that EGFP-TAF-I formed detergent-resistant complexes with genome-bound protein VII. To verify the complex formation with protein VII, we performed the same IF analyses using a TAF-Iβ dimerization mutant TAF-IβPME [25]. Dimerization was previously shown to be critical for cellular and viral chromatin remodeling activity [25,26]. Cells were first transfected with the expression vectors for either EGFP-tagged wildtype TAF-Iβ (WT) or the PME mutant, together with an expression vector for tdiRFP-tagged histone H2B as a transfection marker that was resistant to pre-extraction, and then IF analyses were carried out (Fig 3C). Pre-extraction with infected cells resulted in nuclear puncta of TAF-IβWT but not PME (Fig 3C, third row), confirming a functional interaction with protein VII. As EGFP-TAF-Iβ co-localized with protein VII almost completely (Fig 3B and 3C), our data suggests that the use of this protein allows the rapid detection of Ad chromatin complexes without further antibody staining.

**Fig 4:**
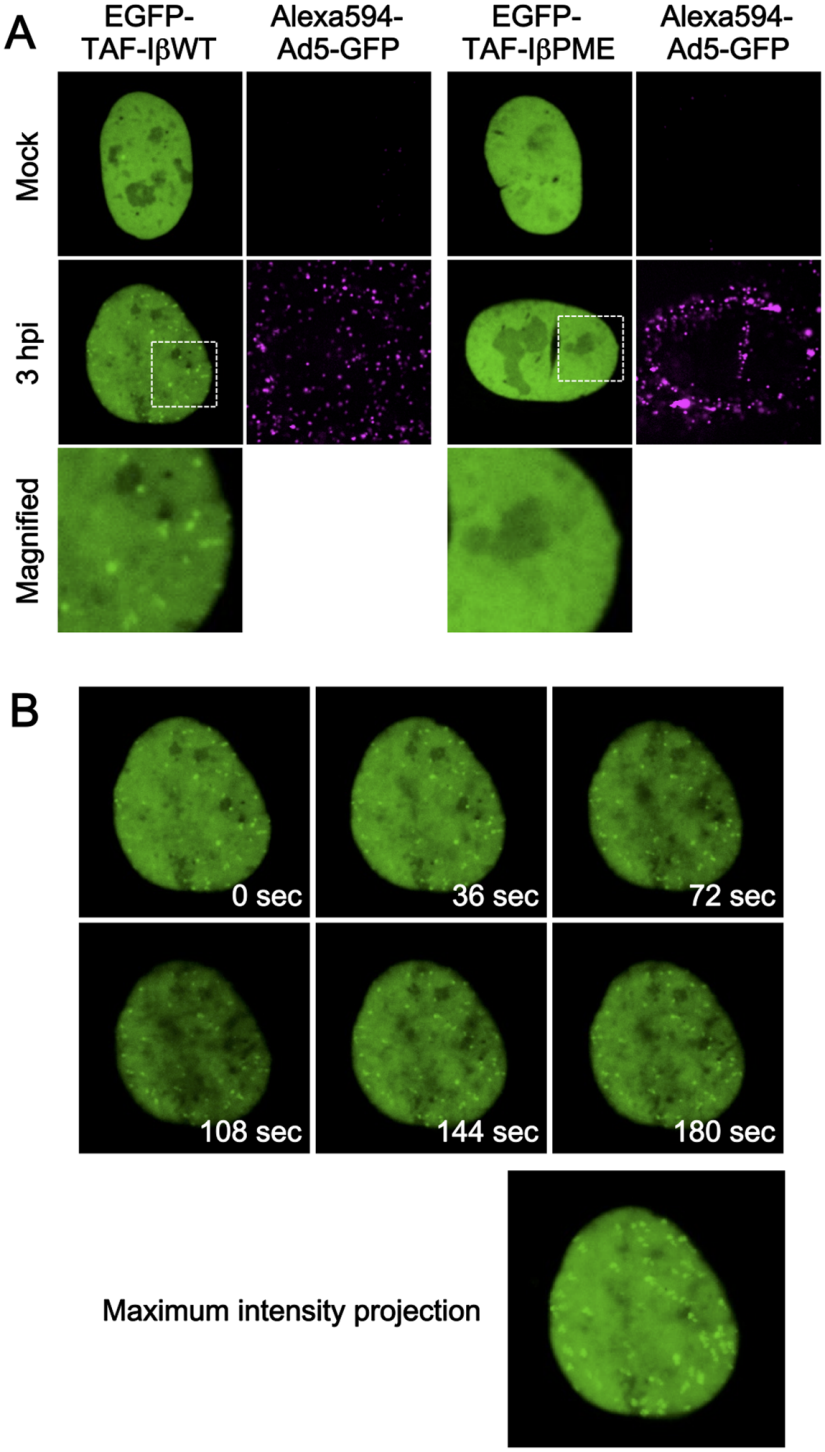
Live-cell imaging using EGFP-TAF-Iβ. (A) Live-cell imaging with EGFP-TAF-Iβ constructs. U2OS cells were transiently transfected with the expression vectors for either EGFP-TAF-IβWT (green, left) or PME mutant (green, right panels) and at 24 hpt were either mock-infected or infected with Alexa594-labeled Ad5-GFP (magenta). At 3 hpi, live-cell imaging was performed, frames were taken every 3 s for 3 min, and snapshots from the movies are shown. Full movies are provided as S1 and S2 Movies for TAF-IβWT and TAF-IβPME, respectively. (B) Series of snapshots of EGFP-TAF-IβWT. Selected images from the time series are shown. An image of maximum intensity projection generated by superimposing the frames of the time series is shown below, showing limited movement of EGFP-TAF-IβWT dots.

Next, we used the EGFP-TAF-Iβ constructs for live-cell imaging (Fig 4 and S1 and S2 Movies). U2OS cells transiently expressing either EGFP-TAF-IβWT or PME were infected with fluorescently labeled viruses, and their movement and subcellular localization was monitored using spinning-disk confocal microscopy. We observed small nuclear puncta that were specific for Ad infection and only appeared in EGFP-TAF-IβWT-expressing cells, similar to what we observed in fixed cells (Fig 4A, second and third rows, and S1 and S2 Movies). This data suggests that detection of individual Ad chromatin complexes in living cells in real-time can be achieved with our method. The mobility of individual Ad chromatin complexes was monitored. As shown in Fig 4B, each TAF-Iβ dot showed confined movement, suggesting that Ad chromatin complexes may be stably associated with specific subnuclear compartments/components, rather than diffusing or actively moving in the nucleus. We noted that although EGFP-TAF-Iβ showed infection-specific puncta after pre-extraction in other cell lines, such as HeLa and H1299 cells, EGFP-TAF-Iβ puncta were less clearly observed in those cells under our live-cell imaging conditions (data not shown). Taken together, these results indicate that fluorescently tagged TAF-Iβ is a functional marker for the localization of incoming Ad chromatin complexes upon nuclear import in living cells.

## Discussion

The conversion of condensed, transcriptionally inactive Ad chromatin into transcriptionally active chromatin at the onset of infection is poorly understood. So far, three major techniques, FISH, genome labeling using nucleoside analogs, and IF using anti-protein VII antibodies, have been used to visualize incoming Ad genome/chromatin complexes. FISH can directly detect viral genomes but only under stringent denaturing conditions. Indeed, simultaneous detection of Ad genomes (by FISH) and protein VII (by IF) has failed [27] or was only partly achieved [10]. Recently, Greber and co-workers reported labeling of Ad genomes with “clickable” nucleoside analogs, which allows viral genome detection under conditions compatible with IF, and demonstrated that the majority of intranuclear viral genomes are protein VII-positive [12]. The authors also showed the presence of small population of protein VII-negative genomes both in the nucleus and cytoplasm [12]. Thus, this method allows direct visualization of viral genomes overcoming some disadvantages of FISH, but requires production and purification of analog-labeled viruses. In contrast, IF assays using anti-protein VII antibodies can be also carried out under mild conditions but do not require pre-treated/labeled viruses. Furthermore, our novel technique using fluorescently labeled TAF-I allows the visualization of incoming viral chromatin complexes without any antibody and can be applicable to not only fixed but also living cells. The major drawback of these methods is the inability to detect protein VII-negative genomes, which can be observed using “clickable” viruses [12]. Nevertheless, several lines of evidence strongly suggest that protein VII- (and TAF-I-) positive genomes are a major and transcription-competent form in infected cells. Firstly, we have demonstrated the functional roles of protein VII and TAF-I in the regulation of viral gene expression [6,16]. Secondly, our previous ChIP assays and the extraction experiments in this study revealed the stable association of both proteins with viral genomes throughout early phases of infection [5,6]. Therefore, we suggest that either IF with anti-protein VII antibodies or the method presented in this study can be used to visualize “functional” chromatin complexes. It is currently unclear how protein VII interacts with viral genomes. It is intriguing to speculate that the primary genome sequence may affect protein VII binding. In this respect, the combination of our methods with genome-modified viral vectors (e.g. high-capacity vectors deleted of large parts of the viral genome) may provide novel insight into viral chromatin dynamics.

In summary, in this study we present a novel antibody against protein VII and an imaging system that is applicable to both fixed and living cells. We believe that these tools provide a significant, easy-to-use technological advancement to better understand Ad chromatin regulations during early phases of infection.

## Supporting Information

S1 Dataset Raw IF images shown in Fig 2. (ZIP)

S2 Dataset Raw IF images shown in Fig 3. (ZIP)

S1 Fig Uncropped image of western blotting shown in Fig 1. (TIFF)

S1 Movie Movie of live-cell imaging using EGFP-TAF-IβWT. U2OS cells were transiently transfected with the expression vector for EGFP-TAF-IβWT and at 24 hpt were either mock-infected (upper panels) or infected with Alexa594-labeled Ad5-GFP (lower panels). At 3 hpi, live-cell imaging was performed as described in Materials and Methods. Frames were taken every 3 sec for 3 min. EGFP proteins and labeled viruses are shown in green (left) and magenta (right panels), respectively.
(AVI)

S2 Movie Movie of live-cell imaging using EGFP-TAF-IβPME. Live-cell imaging was carried out as described for S1 Movie, but using the mutant EGFP-TAF-IβPME instead.
(AVI)
