# Supplementary figures and images for: A Method for Visualization of Incoming Adenovirus Chromatin Complexes in Fixed and Living Cells
Source: PLoS One. 2015 Sep 2;10(9):e0137102. doi: 10.1371/journal.pone.0137102 (PMC4557953; doi:10.1371/journal.pone.0137102)

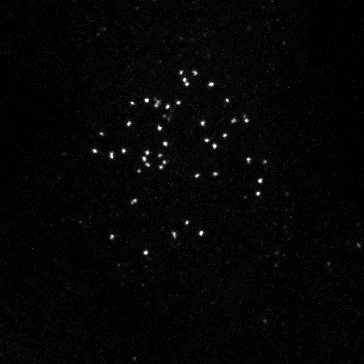

Supplement: S1 Dataset — (ZIP) [file pone.0137102.s001.zip › Komatsu_et_al_Live_Fig2_RAW/Komatsu_Live_Fig2A_3hpi.tif]

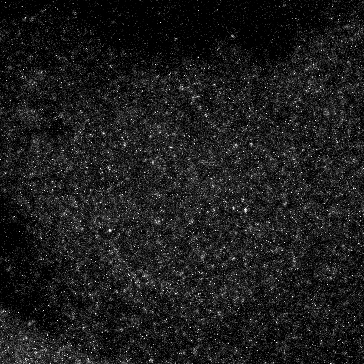

Supplement: S1 Dataset — (ZIP) [file pone.0137102.s001.zip › Komatsu_et_al_Live_Fig2_RAW/Komatsu_Live_Fig2A_MOCK.tif]

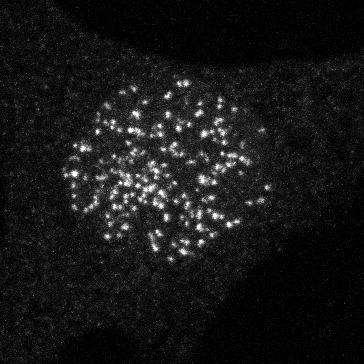

Supplement: S1 Dataset — (ZIP) [file pone.0137102.s001.zip › Komatsu_et_al_Live_Fig2_RAW/Komatsu_Live_Fig2B_3hpi.tif]

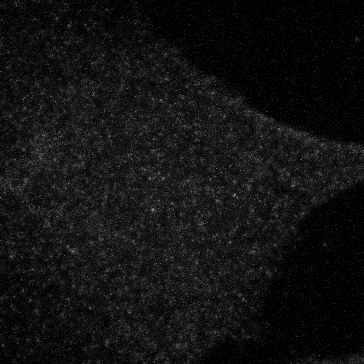

Supplement: S1 Dataset — (ZIP) [file pone.0137102.s001.zip › Komatsu_et_al_Live_Fig2_RAW/Komatsu_Live_Fig2B_MOCK.tif]

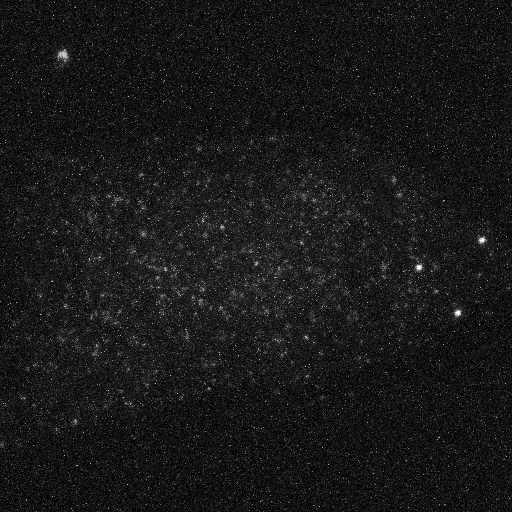

Supplement: S1 Dataset — (ZIP) [file pone.0137102.s001.zip › Komatsu_et_al_Live_Fig2_RAW/Komatsu_Live_Fig2C_0mpi.tif]

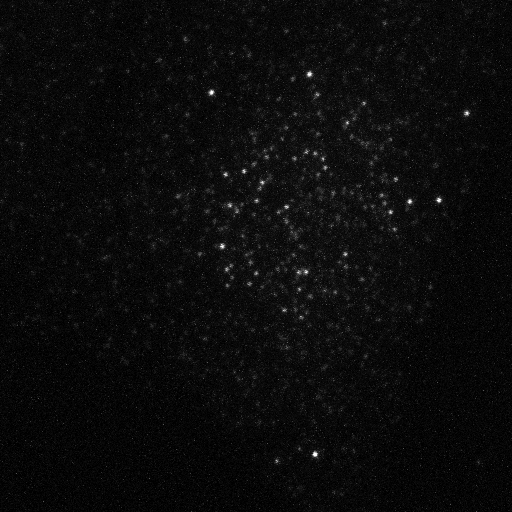

Supplement: S1 Dataset — (ZIP) [file pone.0137102.s001.zip › Komatsu_et_al_Live_Fig2_RAW/Komatsu_Live_Fig2C_20mpi.tif]

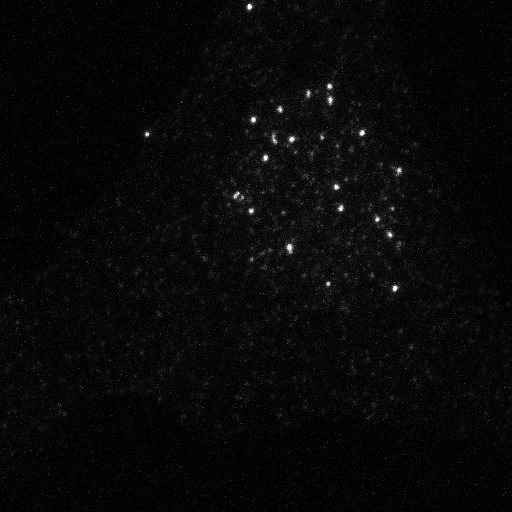

Supplement: S1 Dataset — (ZIP) [file pone.0137102.s001.zip › Komatsu_et_al_Live_Fig2_RAW/Komatsu_Live_Fig2C_60mpi.tif]

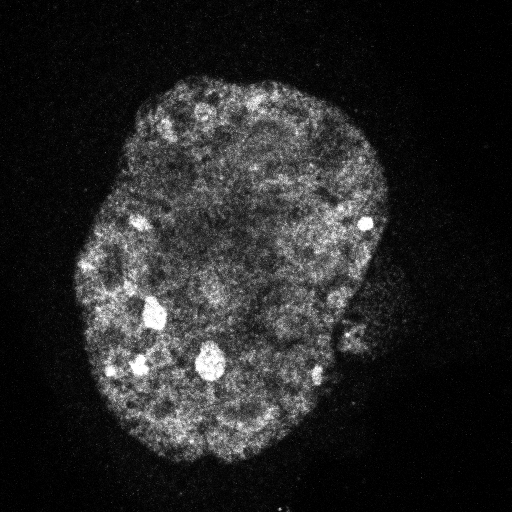

Supplement: S1 Dataset — (ZIP) [file pone.0137102.s001.zip › Komatsu_et_al_Live_Fig2_RAW/Komatsu_Live_Fig2D_left_24hpi.tif]

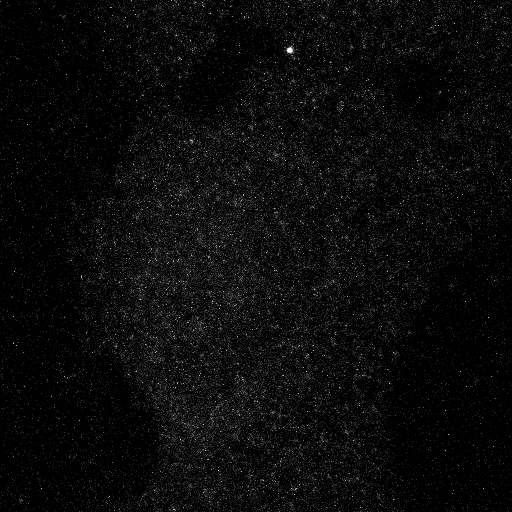

Supplement: S1 Dataset — (ZIP) [file pone.0137102.s001.zip › Komatsu_et_al_Live_Fig2_RAW/Komatsu_Live_Fig2D_left_MOCK.tif]

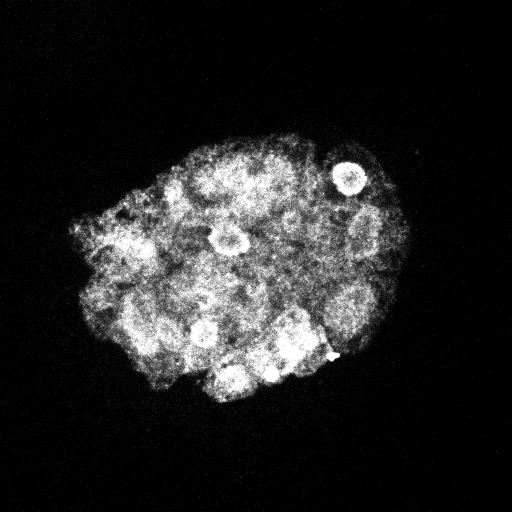

Supplement: S1 Dataset — (ZIP) [file pone.0137102.s001.zip › Komatsu_et_al_Live_Fig2_RAW/Komatsu_Live_Fig2D_right_24hpi.tif]

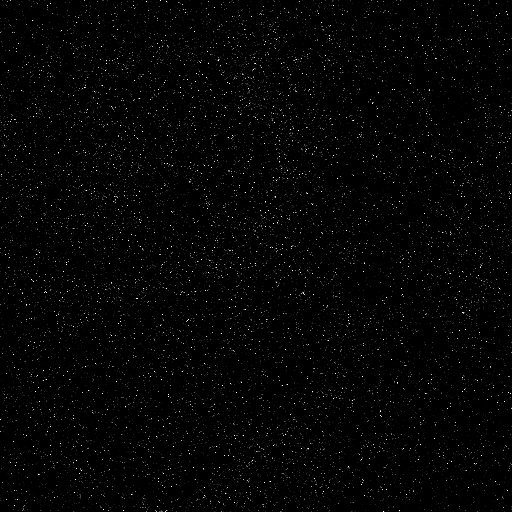

Supplement: S1 Dataset — (ZIP) [file pone.0137102.s001.zip › Komatsu_et_al_Live_Fig2_RAW/Komatsu_Live_Fig2D_right_MOCK.tif]

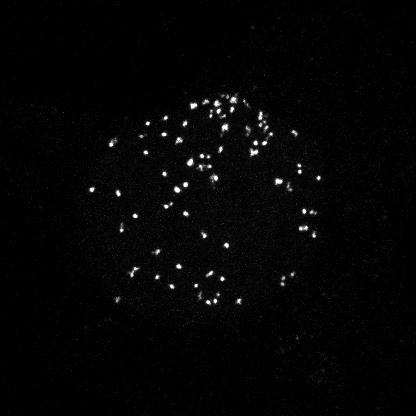

Supplement: S2 Dataset — (ZIP) [file pone.0137102.s002.zip › Komatsu_et_al_Live_Fig3_RAW/Komatsu_Live_Fig3A_3hpi.tif]

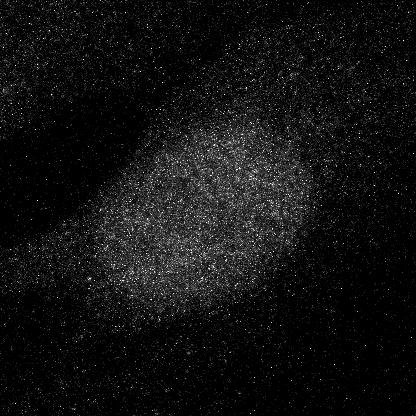

Supplement: S2 Dataset — (ZIP) [file pone.0137102.s002.zip › Komatsu_et_al_Live_Fig3_RAW/Komatsu_Live_Fig3A_MOCK.tif]

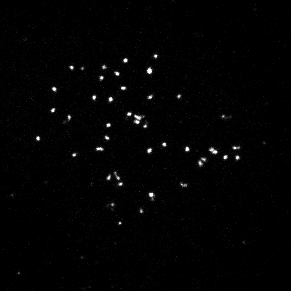

Supplement: S2 Dataset — (ZIP) [file pone.0137102.s002.zip › Komatsu_et_al_Live_Fig3_RAW/Komatsu_Live_Fig3B_3hpi.tif]

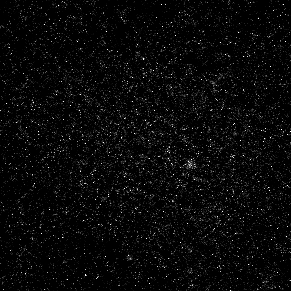

Supplement: S2 Dataset — (ZIP) [file pone.0137102.s002.zip › Komatsu_et_al_Live_Fig3_RAW/Komatsu_Live_Fig3B_MOCK.tif]

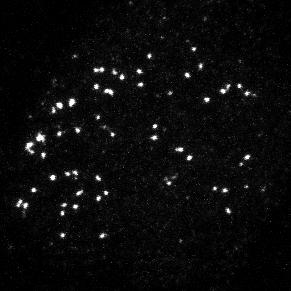

Supplement: S2 Dataset — (ZIP) [file pone.0137102.s002.zip › Komatsu_et_al_Live_Fig3_RAW/Komatsu_Live_Fig3B_Tri3hpi.tif]

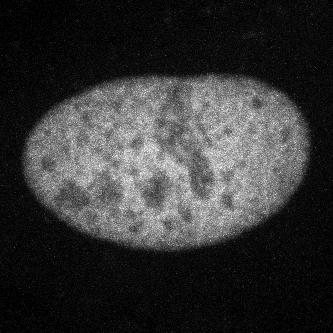

Supplement: S2 Dataset — (ZIP) [file pone.0137102.s002.zip › Komatsu_et_al_Live_Fig3_RAW/Komatsu_Live_Fig3C_PME_MOCK.tif]

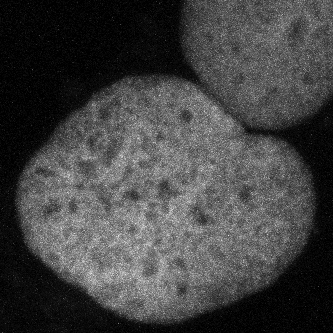

Supplement: S2 Dataset — (ZIP) [file pone.0137102.s002.zip › Komatsu_et_al_Live_Fig3_RAW/Komatsu_Live_Fig3C_PME_Tri3hpi.tif]

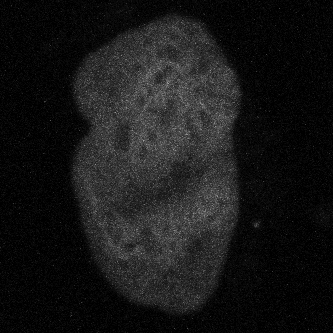

Supplement: S2 Dataset — (ZIP) [file pone.0137102.s002.zip › Komatsu_et_al_Live_Fig3_RAW/Komatsu_Live_Fig3C_PME_TriMOCK.tif]

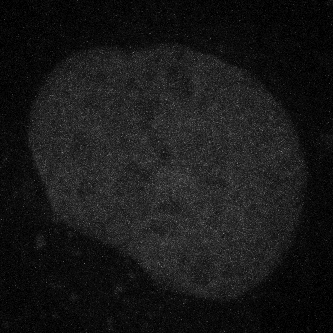

Supplement: S2 Dataset — (ZIP) [file pone.0137102.s002.zip › Komatsu_et_al_Live_Fig3_RAW/Komatsu_Live_Fig3C_WT_MOCK.tif]

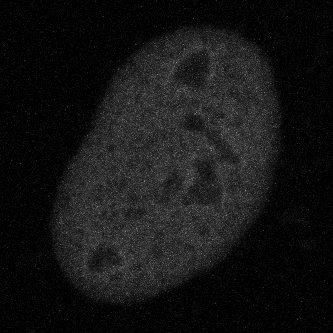

Supplement: S2 Dataset — (ZIP) [file pone.0137102.s002.zip › Komatsu_et_al_Live_Fig3_RAW/Komatsu_Live_Fig3C_WT_Tri3hpi.tif]

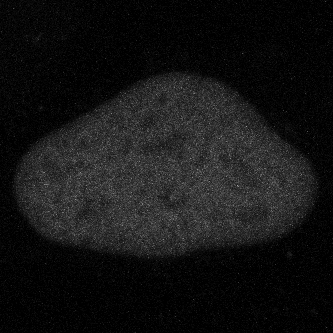

Supplement: S2 Dataset — (ZIP) [file pone.0137102.s002.zip › Komatsu_et_al_Live_Fig3_RAW/Komatsu_Live_Fig3C_WT_TriMOCK.tif]

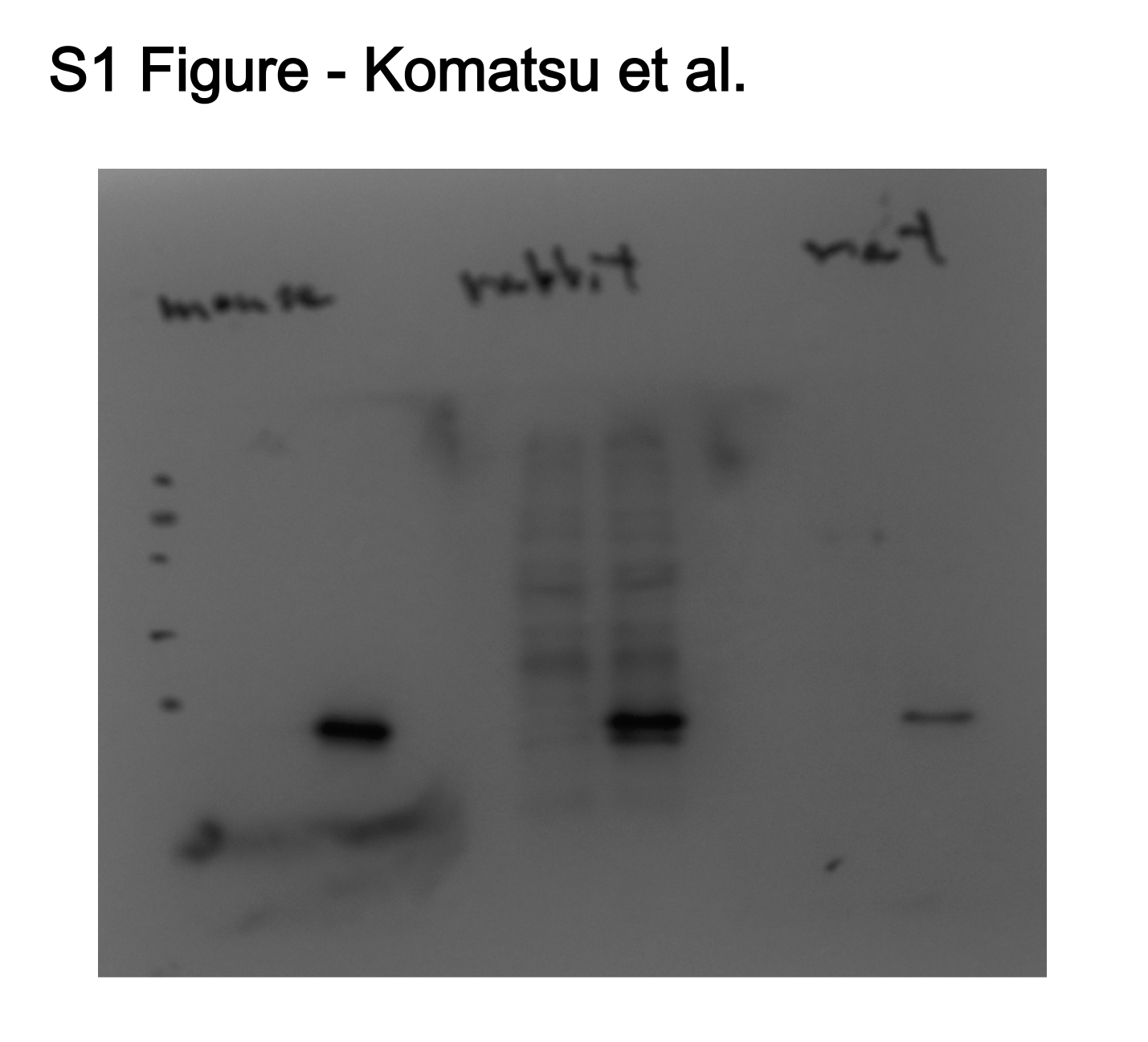

Supplement: S1 Fig — (TIFF) [file pone.0137102.s003.tiff]
